# Knowledge and experience sharing practices among health professionals in hospitals under the Addis Ababa health bureau, Ethiopia

**DOI:** 10.1186/1472-6963-14-431

**Published:** 2014-09-24

**Authors:** Mulusew Andualem Asemahagn

**Affiliations:** Institute of Public Health, University of Gondar, Gondar, Ethiopia

**Keywords:** Knowledge, Experience, Knowledge sharing, Health professionals, Determinants, Ethiopia

## Abstract

**Background:**

Health professionals need updated health information from credible sources to improve their knowledge and provide evidence based health care services. Various types of medical errors have occurred in resource-limited countries because of poor knowledge and experience sharing practices among health professionals. The aim of this study was to assess knowledge-sharing practices and determinants among health professionals in Addis Ababa, Ethiopia.

**Methods:**

An institutional based cross-sectional study was conducted among 320 randomly selected health professionals from August12-25/2012. A pretested, self-administered questionnaire was used to collect data about different variables. Data entry and analysis were done using Epi-Info version 3.5.4 and SPSS version20 respectively. Descriptive statistics and multivariate regression analyses were applied to describe study objectives and identify the determinants of knowledge sharing practices respectively. Odds ratio at 95% CI was used to describe the strength of association between the study and outcome variables.

**Results:**

Most of the respondents approved the need of knowledge and experience sharing practices in their routine activities. Nearly half, 152 (49.0%) of the study participants had knowledge and experience sharing practices. A majority, 219 (70.0%) of the respondents showed a willingness to share their knowledge and experiences. Trust on others’ knowledge, motivation, supportive leadership, job satisfaction, awareness, willingness and resource allocation are the determinants of knowledge and experience sharing practices. Supportive leadership, resources, and trust on others’ knowledge can enhance knowledge and experience sharing by OR = 3.12, 95% CI = [1.89 - 5.78], OR = 2.3, 95% CI = [1.61- 4.21] and OR = 2.78, 95% CI = [1.66 - 4.64] times compared with their counterparts respectively.

**Conclusion:**

Even though most of the respondents knew the importance of knowledge and experience sharing practices, only a limited number of respondents practiced it. Individual, organizational and resource related issues are the major determinants of low knowledge sharing practices. Improving management, proper resource allocation, motivating staffs, and accessing health information sources are important interventions to improve the problem in the study area.

## Background

The rising interest in considering knowledge as a critical asset of health care organizations and its management is becoming an important issue [1, 2]. The World Health Organization (WHO) and other evidences defined knowledge management as “a set of principles, tools and practices that enable people to create, share, translate and apply knowledge to create value and improve effectiveness [3–6].” Health care knowledge sharing can be characterized as the clarification and dissemination of updated health information to staffs, decision makers and other sectors through interactive communication media [6–8].

Knowledge management and experience sharing practices can help health professionals to update themselves and deliver quality health care services [9, 10]. Health professionals can share their knowledge and experiences through lecturing, questioning and answering, demonstration, discussion, internet, video and audio conferences [11–13]. Health professionals can access health information from the two basic health information resources (HIRs): formal (hard and soft copies) and informal (human resources) [14, 15].

Health professionals need up-to-date health information from credible sources to improve their knowledge and provide evidence based healthcare services to their clients [16]. As shown by various studies, developing knowledge sharing habits within the organizations is essential for the success of health institutions and their customers by increasing intellectual capital, reducing costs, and making individuals and organizations competitive in their environment [10, 16–20]. Knowledge sharing practices can be at individual or organization level [21].

Even though the importance of knowledge and experience sharing practices are mentioned by various studies, they are poorly practiced in hospitals of resource-scared countries [22–26]. Health professionals from resource-limited countries are known for their limited information sharing practices [22, 23]. The absence of this crucial issue in hospitals is an important cause for the occurrence of various medical errors such as severe injury, missing-diagnosis, wrong treatment, increased multi drug resistance and unexpected deaths [1, 24–29].

As indicated by different studies from Ethiopia, information and experience sharing practice of health professionals is poor due to several reasons. Health care workers in most of the health institutions are working simply by referring to their handouts and remembering their school trainings [26, 27].

Some of the reasons are poor infrastructure for information sharing, poor health personnel initiation, poor peer education, poor management, absence of internet services and poor information sharing culture among staffs [22, 23, 26, 27]. The presence of knowledge gaps, a competitive environment, government needs, and questions from patients pushed health professionals to have up-to-date health information and experiences [22, 26, 27].

Generally, factors affecting information sharing practices can be grouped as intrinsic (individual) and extrinsic (organizational and technological factors) [30–32]. Some of the individual level factors were time shortage, lack of confidence, knowledge gap, age, culture, poor readiness, language barriers, gender differences, personal initiation and differences in educational status [30–32]. Major organizational factors are management problem, absence of information sources, poor attention from staffs, poor information sharing culture, resource shortage and poor infrastructure [30, 32]. Accuracy, maintenance issues, feasibility, interoperability issues, system failure, acceptance and user-friendliness of the system are major technological related factors [31, 32].

Based on the study findings from Addis Ababa, Ethiopia [23], high turnover of experienced health professionals, absence of retirement, external transfer, death, and personal reasons were also important determinants for the presence of poor knowledge and experience sharing practice. Another study from Bahir Dar, Ethiopia [22] showed that poor staffs’ engagement on knowledge sharing, health information sharing mechanisms shortage, poor infrastructure, poor organizational motivation/support, poor resource allocation and limited communication channels were determinants for knowledge and experience sharing practice.

The aim of this study is to determine the level of knowledge and experience sharing practices among health professionals and to identify determinant factors in hospitals under Addis Ababa Health Bureau (AAHB). Since there is inadequate evidence on this topic in the study area, findings of this study will serve as important evidence for health administrators, policy makers, health professionals, NGOs and researchers to plan and make interventions to improve knowledge and experience sharing practices in the study area.

## Methods

An institutional based cross sectional study was conducted to determine knowledge and experience sharing practice, and associated factors among health professionals working at the five public hospitals under the AAHB from August12-25/2012. Addis Ababa is the capital city of Ethiopia with a population of 2, 738, 248 [33]. The city has ten administrative sub cities and 99 Kebeles. There are 38 hospitals (ten public and 28 NGO and private) in the study area. Of these, AAHB owns only five. There are about 27 governmental owned health centers, 19 higher and 103 medium private clinics [34]. During the study period, there were about 1200 health professionals from different departments working in the five AAHB hospitals.

All health professionals who are the employees of hospitals under AAHB were the source population for this study. The sample size of the study was determined using Epi Info version 7, by taking the total population N = 1200. The knowledge sharing practice of health professionals (p) = 50% since there was no previous study there with the precision error (d) =0.05 at a 95% confidence level and 10% contingency. Thus, the actual sample size for the current study was 291 + 29 = 320.

**Figure 1:**
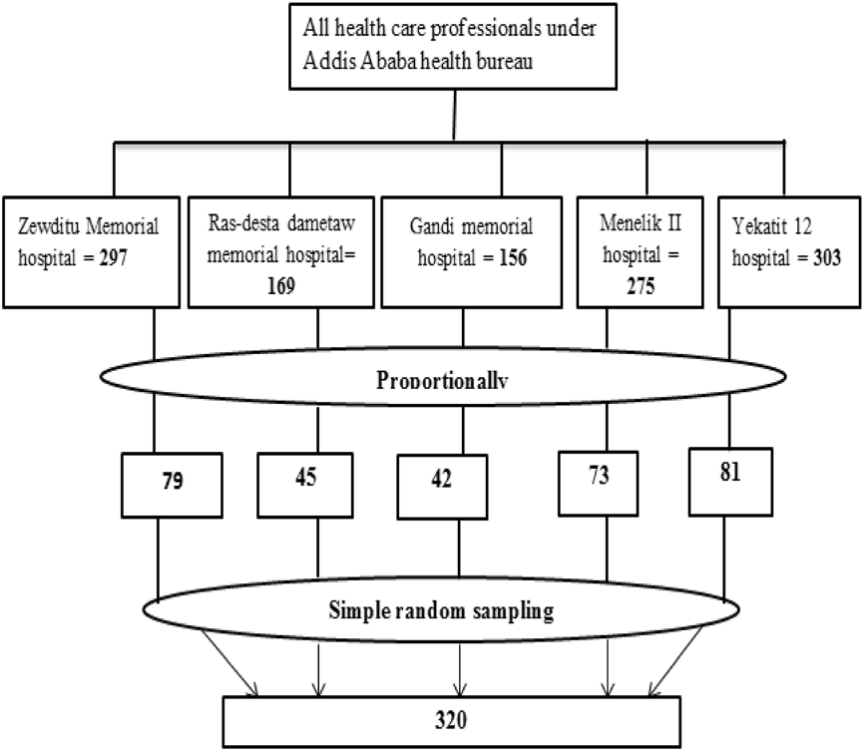
**Sampling procedures from hospitals under Addis Ababa health bureau, 2012.**

There were 279, 169, 156, 275 and 303 health professionals in Zewditu memorial hospital, Ras-Desta Damtew memorial hospital, Gandi memorial hospital, MinillikII hospital and Yekatit12 hospital respectively. The ample size for each hospital was determined proportionally and each sample from each hospital was selected randomly from alphabetical health professionals lists (Figure 1).

Data from the study participants were collected using a pretested self-administered questionnaire. The questionnaire was developed by referring related studies from different sources [11, 26, 35–37]. The tool contained questions related to socio-demographic characteristics, information and experience sharing practices, and factors affecting information sharing of health professionals. The questionnaire was prepared in English, translated into Amharic (local language) and back to English using language experts to check its consistency. Names of the study participants were excluded from the questionnaire to assure data confidentiality. The validity of the questionnaire was checked by conducting pre-test on about 10% of the tool at St. Paulos specialized hospital, which is having the same setups as the study hospitals.

A one day training on the objective of the study, data confidentiality, respondents’ right, informed consent, and data collection techniques was given to the three data collectors and two supervisors prior to the data collection period.

Ethical clearance for this study was obtained from Addis Ababa University Medical Faculty Review Committee. Letter of support was obtained from Addis Ababa administrative health bureau. In addition, informed consent was taken from the heads of each hospital and respondents after explaining the purpose, duration, and required samples.

The data collectors informed health professionals about the purpose of the study, the questionnaire filling process and data confidentiality while distributing the questionnaire and obtained informed consent from all participants. The supervisors and the principal investigator closely supervised the data collectors, example about their approach to the respondents, communication with respondents, responsiveness of data collectors, time utilization, data confidentiality issues and checking data completeness.

The investigator checked all data for completeness and consistency before data entry. Epi-Info version 3.5.4, and SPSS version 20 were used to clean and analysis of data respectively. Descriptive statistics were used to describe the study population in relation to relevant variables. Binary logistic regression was computed to see the effect of each study variable on the outcome variable. Variables with p-value of < 0.2 were subjected in a multivariate logistic regression analysis to evaluate the consistency of the effect after adjusting other variables. The reason of taking variables with p-value < 0.2 is to not miss variables, which may have an impact on the outcome variables. The strength of associations was described using Odds ratio (OR) and a 95% CI.

In general, the causal model approach with forward fitting was used, since the main objective of the study is to identify the potential factors for the presence of poor knowledge and experience sharing in the study area.

## Results

### Socio-demographic characteristics of the study subjects

Three hundred twenty self-administered questionnaires were distributed among the study participants. Of the total distributed questionnaires, 311 (97.0%) were completed and returned back for analyses. Among those respondents, 189 (61.0%) were females. The majority of the respondents (67.0%) were in the age group of 21–30 years (Table 1). More than half of the respondents, 199 (64.0%) have a first degree and above in educational status. By profession, 181 (58.0%) were nurses, 37 (12.0%) were medical laboratory personnel, 29 (10.0%) were pharmacy personnel, 28 (9.0%) were general practitioners, 16 (5.0%) were health officers and the rest 19 (6.0%) were health workers from other categories (Table 1).

**Table 1:** **Socio demographic characteristics of health professionals among selected hospitals in Addis Ababa, 2012**

Study variables	Value (%)
Age in years:	
21-30	208 (67.0%)
31-40	75 (24.0%)
41-50	22 (7.0%)
>50	6 (2.0%)
Sex:	
Male	122 (39.0%)
Female	189 (61.0%)
Educational level:	
Diploma and below	112 (36.0%)
Degree and above	199 (64.0%)
Professional category:	
Nurse	181 (58.0%)
Laboratory personnel	37 (12.0%)
Medical doctor	28 (9.0%)
Pharmacy personnel	29 (10.0%)
Health officers	16 (5.0%)
Other categories	19 (6.0%)
Working experience in years:	
≤10 years	235 (76.0%)
>10 years	76 (24.0%)
Monthly salary in Birr:	
>2000.00	199 (64.0%)
[1500–2000.00]	87 (28.0%)
<1500.00	25 (8.0%)
Job satisfaction:	
Yes	146 (47.0%)
No	165 (53.0%)
Reasons for job dissatisfaction:	
Poor salary	75 (43.0%)
Poor opportunity for updating	52 (30.0%)
Poor recognition and rewarding system	28 (16.0%)
Both poor salary and poor recognition	20 (11.0%)

A large number of respondents (76.0%) have more than five years of professional working experience. Concerning monthly income, 199 (64.0%), 87 (28.0%) and 25 (8.0%) of the respondents have greater than 2000, between and including 1500–2000 and below 1500 Ethiopian Birr respectively (Table 1).

More than half, 165 (53.0%) of the respondents were not satisfied with their job for various reasons. The major causes for their job dissatisfaction were lack of attractive salary 75 (43.0%), poor opportunities for further education 52 (30.0%), lack of performance reward or recognition 28 (16.0%) and 20 (11.0%) were due to both poor salary and absence of proper recognition from their organization (Table 1).

### Motivation, willingness and practices of health professionals to share knowledge

In the case of respondents’ initiation level, 167(54.0%) and144 (46.0%) of the respondents had low and high initiation levels to share their knowledge with their colleagues (Table 2). There were ten questions prepared to assess the initiation level to share knowledge. Those who answered ≥7 questions were considered highly initiated to share their knowledge and experiences. Two hundred nineteen (70.0%) of the respondents have an interest to share their knowledge, experience and skills with their colleagues. On the other hand, 206 (66.0%) of the respondents requested their colleagues to get additional information (Table 2).

**Table 2:** **Initiation, willingness and practice of knowledge sharing among health professionals in Addis Ababa, 2012**

Study variables	Value (%)
Level of initiation to share information:	
Low	167 (54.0%)
High	144 (46.0%)
Willingness of sharing information:	
Yes	219 (70.0%)
No	92 (30.0%)
Asking staffs for information sharing:	
Yes	206 (66.0%)
No	105 (34.0%)
Information and experience sharing:	
Yes	152 (49.0%)
No	159 (51.0%)
ICTs access:	
Yes	68 (22.0%)
No	243 (78.0%)
Major health information sources:	
Books	59 (39.0%)
Trainings	44 (29.0%)
Workshops	32 (21.0%)
Guidelines	17 (11.0%)
Presence of Health information sources:	
Yes	90 (29.0%)
No	221 (71.0%)
Presence of periodic meeting for information sharing:	
Yes	168 (54.0%)
No	143 (46.0%)
Presence of supportive leadership:	
Yes	101 (32.0%)
No	210 (68.0%)
Resource allocation for knowledge sharing:	
Yes	109 (35.0%)
No	202 (65.0%)

Nearly half, 152 (49.0%) of the study participants shared health information (disease information, patient diagnosis and management) and professional experience with their colleagues when needed.

Major health information sources for those who had experience in information sharing were workshops (21.0%), trainings (29.0%), books (39.0%) and guidelines (11.0%). The majority, 219 (70.0%) of the respondents stated that they are willing to share their knowledge and experience with their colleagues. The presence of supportive leader ship and resource allocation for knowledge sharing was reported from 101 (32.0%) and 109 (35.0%) respectively (Table 2).

The majority (71.0%) of the respondents disagreed on the presence of adequate health information resources (books, workshops, trainings, peer education, library, and seminars) within and around their organization. About 54.0% of all study participants agreed that there are no periodic meetings for knowledge sharing within their hospital. About 71.0% of the respondents reported the absence of adequate and updated HIRs in the study area. The absence of information communication technologies (ICTs) within the hospitals was reported from about 243 (78.0%) of the respondents (Table 2).

### Information sharing mechanisms

**Figure 2:**
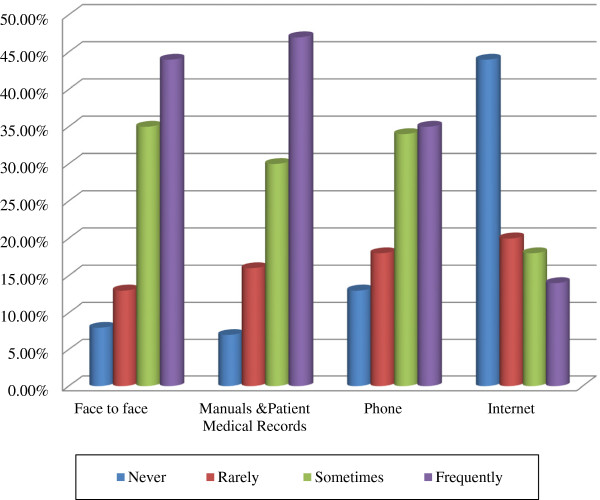
**Information sharing mechanisms among health professionals, Addis Ababa, 2012.**

Nearly half (49.0%) of the respondents used various types of mechanisms to share their knowledge and experiences with their colleagues. Some of them were face-to-face, manuals, patient medical record system, reports, phone and internet. In the case of face to face, 67 (44.0%), 53 (35.0%), 20 (13.0%) and 12 (8.0%) of the respondents used it frequently, sometimes, rarely and never to share their knowledge and experience respectively. About 72 (47.0%), 45 (30.0%), and 25 (16.0%) of the respondents used manuals and patient medical records frequently, sometimes and rarely to share information respectively (Figure 2).

Only about 53 (35.0%) and 51 (34.0%) of health professionals used their phones to share information frequently and sometimes. The least frequently used knowledge-sharing medium in the study area was the internet. It was accessed by only 21 (14.0%) of the respondents frequently, 28 (18.0%) sometimes and 30 (20.0%) rarely (Figure 2).

### Factors affecting knowledge sharing

Factors affecting knowledge sharing practices of health professionals were assessed in three dimensions: individual, organizational and technological related variables. Trust among staffs, awareness, knowledge level, personal initiation, fear of loss of personal competitiveness, intrinsic and extrinsic motivation were identified factors under the individual dimensions (Table 3).

**Table 3:** **Individual related factors for information sharing in Addis Ababa, 2012**

Study variables	Value (%)
Trust is important for knowledge sharing	
Yes	165 (53.0%)
No	80 (26.0%)
Not certain	66 (21.0%)
Trust on staffs’ knowledge:	
Yes	204 (66.0%)
No	107 (34.0%)
Aware of the importance of knowledge sharing:	
Yes	218 (70.0%)
No	93 (30.0%)
Knowledge sharing reduce competitiveness:	
Yes	62 (20.0%)
No	202 (65.0%)
Not sure	46 (15.0%)
Information sharing time wasting & make busy:	
Yes	112 (36.0%)
No	199 (64.0%)
Intrinsic motivation vital for information sharing:	
Yes	193 (62.0%)
No	45 (14.0%)
Not sure	73 (24.0%)
Presence of intrinsic motivation	
Yes	250 (80.0%)
No	61 (20.0%)
Enjoyed with information sharing	
Yes	211 (68.0%)
No	100 (32.0%)
Extrinsic motivation vital for information sharing	
Yes	250 (80.0%)
No	29 (9.0%)
Not sure	32 (11.0%)
Presence of extrinsic motivation	
Recognition	69 (19.0%)
Financial	27 (5.0%)
Not	215 (76.0%)

Supportive leadership, resource allocation, access of information sources, presence of periodic meetings and infrastructure were commonly identified organizational factors (Table 2). More than half, 165 (53.0%), of the respondents reported that trust among colleagues is important for knowledge and information sharing practices. About 202 (65.0%) of the respondents mentioned that knowledge sharing does not reduce the competitiveness of individuals who share their knowledge and experiences. Around 112 (36%) of the respondents perceived that knowledge and experience sharing is time consuming and makes them busy. The majority, 80% and 62% of the respondents reported that extrinsic and intrinsic motivation can affect the knowledge and experience sharing practices (Table 3).

### Bivariate and multivariate analysis on determinants of knowledge sharing practices of health professionals

In the multivariate logistic regression analysis, work experience, extrinsic motivation, intrinsic motivation, awareness, supportive leader ship, resource allocation, and willingness to share knowledge were positively associated with knowledge and experience sharing practices of health professionals in the study area (Table 4).

**Table 4:** **Association between selected variables and knowledge sharing practices of health professionals in hospitals under Addis Ababa health bureau, 2012**

Variables	Knowledge sharing		COR 95% CI	AOR 95% CI
	Yes N (%)	No N (%)		
Sex:				
Male	62 (20.0%)	60 (19.0%)	1.14 [0.70 - 1.84]	1.09 [0.44 - 1.58]
Female	90 (29.0%)	99 (32.0%)	1	1
Age in years:				
21-30	110 (35.0%)	98 (34.0%)	1.68 [0.95 - 2.98]	1.40 [0.62 - 1.82]
31-40	30 (7.0%)	45 (15.0%)	1.50 [0.63 - 3.57]	1.23 [0.31 - 2.11]
>40	12 (4.0%)	16 (5.0%)	1	1
Educational category:				
Degree and above	105 (34.0%)	94 (30.0%)	1.54 [0.94 - 2.53]	1.80 [0.98 - 3.12]
Diploma and below	47 (15.0%)	65 (21.0%)	1	1
Work experience:				
≤10 years	132 (42.0%)	103 (31.0%)	3.59 [1.96 - 6.63]	2.45 [1.46 - 4.78]
>10 years	20 (6.0%)	56 (18.0%)	1	1
Job satisfaction:				
Yes	78 (25.0%)	68 (22.0%)	1.14 [0.88 - 2.26]	1.12 [0.79 - 2.18]
No	74 (24.0%)	91 (29.0%)	1	1
HIRs access:				
Yes	52 (17.0%)	38 (12.0%)	2.07 [1.22 - 3.51]	1.87 [1.14 - 3.23]
No	88 (28.0%)	133 (43.0%)	1	1
Time availability:				
Yes	54 (17.0%)	58 (19.0%)	1.33 [0.81 - 2.17]	1.20 [0.76 - 2.14]
No	82 (26.0%)	117 (38.0%)	1	1
Extrinsic motivation:				
Yes	56 (18.0%)	40 (13.0%)	2.61 [1.55 - 4.41]	2.42 [1.32 - 4.01]
No	75 (24.0%)	140 (45.0%)	1	1
Trust on staffs’ knowledge:				
Yes	106 (34.0%)	98 (31.0%)	1.43 [0.87 - 2.36]	1.38 [0.82 - 2.20]
No	46 (15.0%)	61 (20.0%)	1	1
Awareness:				
Yes	128 (41.0%)	90 (29.0%)	4.09 [2.32 - 7.26]	3.25 [1.96 -5.92]
No	24 (8.0%)	69 (22.0%)	1	1
Supportive leader ship:				
Yes	67 (22.0%)	34 (11.0%)	2.90 [1.71 - 4.91]	3.12 [1.89 - 5.78]
No	85 (27.0%)	125 (40.0%)	1	1
Intrinsic motivation:				
Yes	133 (43.0%)	117 (38.0%)	2.51 [1.33 - 4.77]	2.10 [1.12 - 3.99]
No	19 (6.0%)	42 (13.0%)	1	1
Resource allocation:				
Yes	65 (21.0%)	44 (14.0%)	1.95 [1.18 - 3.22]	2.31 [1.61 - 4.21]
No	87 (28.0%)	115 (37.0%)	1	1
ICTs access:				
Yes	39 (13.0%)	29 (9.0%)	1.55 [0.87- 2.76]	1.36 [0.62 - 2.51]
No	113 (36.0%)	130 (42.0%)	1	1
Willingness to share knowledge:				
Yes	131 (42.0%)	88 (28.0%)	5.03 [2.79 - 9.14]	4.21 [2.03 - 6.79]
No	21 (7.0%)	71 (23.0%)	1	1

Respondents having work experience of ≤10 years shared their knowledge and experience to their colleagues 3.59 [1.96-6.63] times more often than those with a working experience of >10 years. The presence of HIRs can enhance knowledge and experience sharing by 2.07 [1.22-3.51] times compared with HIRs shortage. Respondents who have extrinsic and intrinsic motivation performed knowledge sharing 2.61[1.55-4.41] and 2.51 [1.33-4.77] times more often than their counter parts respectively. Respondents who have supportive leader ship from their hospitals can share their knowledge 2.90 [1.71-4.91] times more often to their colleagues more than those who do not have a supportive leadership. Respondents who have awareness on knowledge sharing shared their knowledge 4.09 [2.32-7.26] times more compared with their counter parts (Table 4).

## Discussion

This institution-based study attempted to assess the knowledge sharing practices of health professionals and associated factors in Addis Ababa, Ethiopia. A majority, 218 (70.0%) of health professionals acknowledged the importance of the presence of knowledge sharing practices in their hospitals. This finding was also supported by various studies at different times [4, 9, 10, 20, 21, 28, 38]. Even though, most of the respondents have knowhow on the importance of knowledge and experience sharing, only 152 (49.0%) of them actually shared their knowledge and experiences to their colleagues. This finding is higher compared with study findings from Bahir Dar, Ethiopia [22] where only 19.0% of the hospital staffs shared their knowledge and experience with their colleagues. The most possible reasons for the difference might be the difference in infrastructure, staffs awareness, management support, resource allocation, and geographical location.

In this study, 219 (70.0%) of the respondents were aware and expressed their willingness to share knowledge, and experience with their colleagues. This finding is relatively larger compared to study findings from Ethiopia [22] where about 52.0% of the health professionals were willing and aware about knowledge sharing. The probable reasons for this variation could be differences in infrastructure, staffs’ initiation, access of ICTs and staff’s attitude. Regarding the personal initiation to share knowledge and experiences, only 54.0% showed personal initiation. This may be due to the presence of poor access to information resources (17%), poor ICTs (22.0%), resource limitation (65.0%), job dissatisfaction (56.0%) and poor supportive leadership (67.0%) in the study area.

Mostly accessed HIRs in the study area were medical textbooks 59 (39.0%), trainings 44 (29.0%), workshops 32 (21.0%) and guidelines 32 (21.0%). These were also mentioned as major HIRs for health professionals in other studies [14–16, 35, 38]. About 206 (66.0%) of the study participants had experiences of asking their senior staffs to get knowledge and experience. This is different from study findings in Addis Ababa [27], where the major HIRs were protocol manuals 115 (39.0%), text books 84 (25.0%) and in-service trainings 211(62.0%). The current finding is slightly lower compared to study findings from Uganda [35] where frequently accessed HIRs were discussions with colleagues (89.0%) and textbooks (77.0%). Major reasons for being lower in this study may be less attention from staffs, fear of criticism, poor readiness, trust on staffs’ knowledge and absence of adequate HIRs in the study area.

More than half, 65.0% of the respondents believed that knowledge sharing could increase the competitiveness of health professionals. This is strongly supported by various studies [29, 30, 36, 37, 39, 40] since information is the most valuable source to update health professionals’ knowledge and deliver quality health care services. Such situations can create positive competitions among staffs and even among hospitals since their quality services will attract more customers. Only 20.0% of the study participants believed that information sharing will waste their time and make them busy. This may be due to the absence of HIRs in the study area, so that it will take much time to search and make them not ready to update and share to others.

Around 62.0% and 80.0% of health professionals agreed upon the importance of internal and external motivation for effective knowledge sharing practices among the staffs respectively. It is true that if health professionals are motivated and aware of knowledge sharing, they will increase this habit. This also supported by different study findings from different areas [31, 32, 41]. Even though recognition letters and finances are very essential and common means to motivate staffs in most of the organizations, they were poorly practiced (recognition 19.0% and finance 5.0%) in the study area. This might be due to the presence of poor resource allocation (35.0%), poor supportive leadership (33.0%) and high job dissatisfaction among staffs (56.0%) in the study area. These reasons were also reasons in other studies [30, 42, 43].

As also stated by other studies [15, 44], ICTs became backbones for health care institutions in this competing environment. Nowadays, various stakeholders have given attention to the application of ICTs in health care facilities to deliver evidence based quality health care services [15, 31, 44]. However, the opposite was true in the study areas. The majority, 243 (78.0%) of the health professionals reported the presence of poor ICTs access in the study area. This is also supported by study findings from Bahir Dar [22] and Addis Ababa [27, 45]. The most possible reasons could be resource limitation, infrastructure, poor attention from management and staffs and lack of skilled personnel.

Identifying the most important factors affecting knowledge sharing practices among hospital staffs is very essential for managers, health professionals and other concerned bodies in order to make evidence-based plans to solve the problem.

The most important identified determinants for the presence of low knowledge and experience sharing practice in the study area are working experience, motivation (internal and external), awareness on knowledge sharing, HIRs access, supportive leadership, resource allocation and staffs’ willingness to share knowledge (Table 4). These were also mentioned as determinant factors for information sharing practice of health professionals in other studies [22, 30, 32, 41–43, 45].

## Conclusion

Even though most of the health professionals from resource-limited countries are willing and have an intrinsic motivation to share their knowledge and experiences, they practiced it poorly for several reasons. The identified main determinants are access to HIRs, motivation (intrinsic and extrinsic), working experience, supportive leadership, resource allocation, staff’s awareness and willingness to share knowledge. Improving management activities, resource allocation, HIRs access and staffs’ motivation are important interventions to improve the problem among the health professionals. Exploring the mechanisms of increasing knowledge and experience sharing practices is a future research agenda in the study area.

## References

[1] Abidi S (2001). Knowledge management in healthcare: towards ‘knowledge-driven’ decision -support services. Int J Med Inform.

[2] Anca M (2008). Knowledge dynamics. Revista Informatics Econ.

[3] Nonaka I, Toyama R, Byosière P (2001). A theory of organizational knowledge creation: understanding the dynamic process of creating knowledge. Handbook of organizational learning and knowledge.

[4] WHO (2006). Technical paper on regional strategy for knowledge management to support public health.

[5] Eid M, Nuraddeen A: *The impact of learning culture and information technology use on knowledge sharing: 17th European conference on information systems*. Available at http: http://www.ecis 2009.it/papers/ecis2009–0037

[6] Veronique L (2010). Integrated knowledge translation for globally oriented public health practitioners and scientists: framing together sustainable transferontier knowledge translation vision. J Multidisciplinary Health care.

[7] Ballantyne D (2004). Dialogue and its role in the development of relationship specific knowledge. J Business Ind Mark.

[8] Tsoukas H, Vladimirou E (2001). What is organizational knowledge. J Manag Stud.

[9] Ives W, Gordon C: *Knowledge management journey: navigating the IT/HR turf wars*. Available at http://www.accenture.com

[10] Cohen W, Levinthal D (1990). Absorptive capacity: a new perspective on learning and innovation. Adm Sci Q.

[11] Zack MH (1999). Managing codified knowledge. Sloan Manage Rev.

[12] Alavi M, Leidner DE (2001). Knowledge management and knowledge management systems: conceptual foundations and research issues. MIS Q.

[13] Ryu S, Ho S, Han I (2003). Knowledge sharing behavior of physicians in hospitals. Expert Systems Appl.

[14] Wilson TD (2000). Human Information Behavior.

[15] Mohamed A (2011). Information Needs and Information Seeking Behavior of Libyan Doctors Working in Libyan Hospitals [A Doctoral Thesis].

[16] Ghebre HA (2005). Assessment of Health Management Information System in Addis Ababa Health Bureau [MSc Thesis].

[17] Lin C-L (2000). Knowledge Transfer and Management Consulting: A Look at “The Firm”, Business Horizons, January-February.

[18] Nonaka I, Ichijo K, Krogh G (2000). Enabling Knowledge Creation: how to Unlock the Mystery of Tacit Knowledge and Release the Power of Innovation.

[19] Pan S, Scarborough H (1998). A sociotechnical view of knowledge sharing at Buckman Laboratories’. J Knowl Manag.

[20] Zhang J, Faerman S, Cresswell A (2006). The effect of organizational/technological factors and the nature of knowledge on knowledge sharing. The 39th Hawaii International Conference on System Sciences.

[21] Lin HF (2007). “Knowledge sharing and firm innovation capability: an empirical study”. Int J Manpow.

[22] Adem A (2010). Knowledge Sharing Among Health Professionals the Case of Felege Hiwot Referral Hospital Bahirdar.

[23] City Government of Addis Ababa Health Bureau (2011). Report of Human Resource Department.

[24] WHO (2004). Knowledge management strategy.

[25] Ipe M (2003). Knowledge sharing in organizations: a conceptual framework. Hum Resour Dev Rev.

[26] Andualem M, Kebede G, Kumie A (2013). Information needs and seeking behaviour among health professionals working at public hospital and health centres in Bahir Dar, Ethiopia. BMC Health Serv Res.

[27] Lemma I (2009). Assessment of access to health information resources among HPs working in HIV/AIDS and family health units of public health centers in Addis Ababa [MSc thesis].

[28] Moody D, Shanks G (1999). “Using Knowledge Management and the Internet to Support Evidence Based Practice: A Medical Case Study.”.

[29] Dubow J, Chetley A (2011). Improving the Health, Connecting People: The Role of ICTs in the Health Sector of Developing Countries [Internet].

[30] Riege A (2005). Three-dozen knowledge-sharing barriers managers must consider. J Knowl Manag.

[31] Hinds P, Pfeffer J (2003). Why Organizations do not “Know What They Know”: Cognitive and Motivational Factors Affecting the Transfer of Expertise.

[32] Orlikowski W (1993). Learning from notes: organizational issues in groupware implementation. Inf Soc.

[33] FDRE (2008). Summary and Statistical Report of the 2007 Population and Housing Census.

[34] Lemecha G (2008). Personal Communication.

[35] Musoke MG (2000). Information and its value to health workers in rural Uganda: a qualitative perspective [internet]. Health Library Review.

[36] Edejer T (2000). Disseminating health information in developing countries: the role of the internet. BMJ.

[37] Szulanski G (1996). Exploring internal stickiness: impediments to the transfer of practice within the firm. Strateg Manag J.

[38] Kieslowski M (2006). Knowledge management prerequisites for building an information society in health care. Int J Med Inform.

[39] Pan S, Scarborough H (1999). Knowledge management in practice; an exploratory case study. Technol Anal Strateg Manage.

[40] Scarborough H (2003). Why your employees do not share what they know. KM Review.

[41] Ajzen I (1991). The theory of planned behavior. Organ Behav Hum Decis Process.

[42] Hsiu-Fen L (2007). Knowledge sharing and firm innovation capability: an empirical study. Int J Manpow.

[43] Lin HF (2007). Effects of extrinsic and intrinsic motivation on employee knowledge sharing intentions. J Inf Sci.

[44] Ethiopian Federal Ministry of Health, WHO, CSA,HMN (2007). Assessment of the Ethiopian National Health Information System Final Report.

[45] Brhanesilassie E (2009). Physicians’ Culture of use of Online Medical Evidence to Improve Clinical Care of Patients [MSc Thesis].

[CR46] The pre-publication history for this paper can be accessed here: http://www.biomedcentral.com/1472-6963/14/431/prepub

